# Possible variables related to paradoxical findings between PCR and hemoculture assays in rat experimental sepsis

**DOI:** 10.1186/cc12969

**Published:** 2013-11-05

**Authors:** Marcello R da Silva, Marcos M Caseiro, Dercy José de Sá Filho, Ivan HJ Koh

**Affiliations:** 1 Department of Pediatrics, Federal University of São Paulo, Brazil; 2 Department of Pediatrics, Lusíada University Center, Lisbon, Portugal; 3 Department of Surgery, Federal University of São Paulo, Brazil

## Background

A positive blood culture (BC) is considered the gold standard method for the sepsis diagnosis, although its sensibility is low (10 to 30%) which demands a better diagnostic tool to limit broad-spectrum antibiotic use in the majority of patients without culture-based sepsis diagnosis. Besides, after microbial invasion, they can remain live, dead or fragmented in the bloodstream, thus limiting BC efficiency. Herein we evaluated the PCR diagnostic efficacy under live, dead and bacterial DNA contents in the bloodstream.

## Materials and methods

Wistar rats were distributed in three groups (*n *= 20/group) based on live, dead and DNA inoculations. The LPS+DNA group (1 mg/kg LPS injection plus 4 hours later DNA injection, *n *= 10) was designed for DNA detection under an induced inflammatory state. Live, Dead and extracted DNA forms of *Pseudomonas aeruginosa *(ATCC 27853) relative to 2 ml of 10^7 ^colony-forming units/ml were injected into the circulation. Blood samples were collected after 20 minutes and 6 hours (*n *= 10/group/period), and were submitted to nested PCR assay using general and specific primers. BC was performed with 200 μl and 3,000 μl only in the Live group.

## Results

In the Live group, at 20 minutes the sensibility was 100% by both BC and PCR and at 6 hours the sensitivity was 60% (with 200 μl) and 90% (with 3,000 μl) in BC, and 80% in PCR sampled with 50 μl blood volume. In the Dead group, the PCR sensitivity was 90% at 20 minutes and 50% at 6 hours. In the DNA group, the sensitivity remained at 50% independent of time. The inflamed condition did not change PCR sensitivity. Overall data showed that in both techniques the sensitivity dropped with time. In the BC assay the positivity was dependent on sampled blood volume, and in the PCR it was related to live or dead condition. These findings suggest that the live bacteria remain for a short period of time in the bloodstream while DNA can last for longer periods.

## Conclusions

Considering that PCR is performed with 40× less blood compared with a habitual BC, PCR can be an assay of choice when BC is negative and in conjunction in a live bacteria circulating condition. Besides, the PCR assay with specific primers can be a useful method for sepsis diagnosis in specific bacterial surge events in the ICU, thus improving antibiotic usage potentials.

